# Apigenin analogues as SARS-CoV-2 main protease inhibitors: *In-silico* screening approach

**DOI:** 10.1080/21655979.2022.2027181

**Published:** 2022-01-20

**Authors:** Ameny Farhat, Hajer Ben Hlima, Bassem Khemakhem, Youssef Ben Halima, Philippe Michaud, Slim Abdelkafi, Imen Fendri

**Affiliations:** a Laboratory of Plant Biotechnology, Faculty of Sciences of Sfax, University of Sfax, Sfax, Tunisia; b Laboratoire de Génie Enzymatique et Microbiologie, Equipe Biotechnologie des Algues, Ecole Nationale d’Ingénieurs de Sfax, Université de Sfax, Sfax, Tunisia; c Riadi Labs, National School of Computer Science, Manouba University, Manouba, Tunisia; d Institut Pascal, Université Clermont Auvergne, CNRS, Clermont Auvergne INP, Clermont-Ferrand, France

**Keywords:** SARS-Cov-2 main protease, apigenin analogues, docking, inhibitors

## Abstract

The COVID-19 new variants spread rapidly all over the world, and until now scientists strive to find virus-specific antivirals for its treatment. The main protease of SARS-CoV-2 (M^pro^) exhibits high structural and sequence homology to main protease of SARS-CoV (93.23% sequence identity), and their sequence alignment indicated 12 mutated/variant residues. The sequence alignment of SARS-CoV-2 main protease led to identification of only one mutated/variant residue with no significant role in its enzymatic process. Therefore, M^pro^ was considered as a high-profile drug target in anti-SARS-CoV-2 drug discovery. Apigenin analogues to COVID-19 main protease binding were evaluated. The detailed interactions between the analogues of Apigenin and SARS-CoV-2 M^pro^ inhibitors were determined as hydrogen bonds, electronic bonds and hydrophobic interactions. The binding energies obtained from the molecular docking of M^pro^ with Boceprevir, Apigenin, Apigenin 7-glucoside-4’-p-coumarate, Apigenin 7-glucoside-4’-trans-caffeate and Apigenin 7-O-beta-d-glucoside (Cosmosiin) were found to be −6.6, −7.2, −8.8, −8.7 and −8.0 kcal/mol, respectively. Pharmacokinetic parameters and toxicological characteristics obtained by computational techniques and Virtual ADME studies of the Apigenin analogues confirmed that the Apigenin 7-glucoside-4’-p-coumarate is the best candidate for SARS-CoV-2 M^pro^ inhibition.

## 1. Introduction

Coronavirus infection 2019 (COVID-19) has caused more than 237,383,711 confirmed cases and 4,842,716 deaths until the 10 October 2021 in the world (https://covid19.who.int/). Therapies against coronavirus can be classified into two strategies such as drugs acting on the immune system and drugs targeting the virus. Vaccine development has been accelerated, with more than 6,364,021792 vaccine candidates (https://covid19.who.int/). Unfortunately, despite the high level of vaccination and the reduction of the transmission, these therapies may lose their efficiency if the virus mutates and/or changes its antigenicity as observed with the South African variant (variant B.1.351) [1], ‘Epsilon’ variant (B.1.429) in Taiwan [2] and ‘Mu’ variant (B.1.621) in Colombia [3]. The key SARS-CoV-2 targets for therapies comprise a structural protein (responsible for replication, transcription and host cell recognition) and three nonstructural proteins (RdRp, PL^pro^ and 3 CL^pro^) [4]. It was recently found that the main protease of this virus (M^pro^) plays a crucial role in SARS-CoV gene expression and replication [5]. Moreover, genome sequence analyses revealed that COVID-19 shares a high level of sequence similarities with SARS-CoV and MERS-CoV [6]. M^pro^ has been validated as an attractive target for anti-SARS-CoV drug design, and a variety of inhibitors have been developed [7,8], especially considering the re-use of existing MERS and SARS M^pro^ inhibitors. Recently, approved inhibitors including Darunavir [9], Danoprevir [10] and Boceprevir [11] have been used to treat COVID-19 patients. Boceprevir is the recommended treatment as inhibitor for the M^pro^ SARS-CoV-2. However, natural sources such as micro-organisms [12], algae [13] and plants [14] need to be explored to produce new pharmaceutical treatments against SARS-CoV-2. Natural active constituents played a crucial role in drug discovery to treat diverse diseases because of their natural characteristics, lower toxicity and fewer drug remnants in body [15]. Several natural molecules were reported to be able of inhibiting the main protease of Sars-CoV-2 such as quercetin, gallocatechin gallate and epigallocatechin gallate with IC_50_ values of 73 µM, 47 µM and 73 µM, respectively [16,17]. The Apigenin (4′,5,7-trihydroxyflavone), a glycoside from the flavones class, is found in many fruits and vegetables, more particularly in Tunisian plants such as *Retama raetam Forssk* [18], *Zizyphus lotus* L. [19] and in seven principal Tunisian olive varieties [20]. The Apigenin produced from plants have antioxidant [21], anticancer [22–24], anti-inflammatory [25] and anti-hyperglycemic activities [26] and were previously used as compounds in drug discovery. Recent studies have also shown antiviral efficacy of Apigenin against several viruses [26–32]. In addition, Khandelwal et al. (2020) [33] described that Apigenin have antiviral effects against buffalopox virus.

*In-silico* approaches have accelerated the process of drugs finding compared to the conventional methods [34]. Molecular docking has been used to predict the binding models of inhibitors to several targets. They have been successfully used to design or to study the interaction between M^pro^ and promising inhibitors [7,35]. This study was undertaken to investigate the viral adaptation especially the genetic stability of SARS-CoV-2 main proteases from different variants as well as the inhibitory activities of Apigenin and its analogues against this target *via in-silico* studies (binding energies, detailed interactions and pharmacokinetic properties).

## 2. Materials and methods

### 2.1. Amino acid sequence analyses and homology modeling

Sequence analysis (GenBank 6WTT-A [20], QVD51579.1, QWF00346.1, QMV29895.1, QRX05355.1, QTN92506.1, QRW91276.1, QNN90050.1, QNN90062.1 and QNN90074.1) and multiple alignments were performed using the BLAST and CLUSTALW programs [36]. The prediction of the protein secondary structure was performed using the DSSP program [37], while the editing of the alignment including the superimposition of secondary structures was conducted using the ESPript 3.0 program [38]. The automated comparative protein structure homology modeling server, Geno3D (https://geno3d-prabi.ibcp.fr) generated the 3D structure models of SARS-CoV-2 M^pro^ using the published structure as template (PDB-code 6WTT) [39]. PyMOL (http://www.pymol.org) and ViewerLite 5.0 softwares (https://www.3dsbiovia.com) were used to visualize and analyze the generated model structures and to construct the graphical presentations and illustrative figures.

### 2.2. Docking methodology

The three-dimensional x-ray crystal structure of SARS-CoV-2 M^pro^ (pdb code: 6WTT) was retrieved in pdb format from Protein Data Bank with resolution 2.15 Å [39]. After that, the co-crystallized ligand of the SARS-CoV-2 M^pro^ structure was extracted. Then, it was prepared in AutodockVina by removal of water and solvent molecules, addition of polar hydrogens, removal of the bound ligand and partial charge assignment and saved as.pdbqt format using AutodockVina to be included as a reference in the virtual screening. The grid box was defined by selecting the co-crystallized inhibitors to keep the center of each docked Apigenin analogues with same dimensions of binding box. Moreover, the grid box center was adjusted X = 4. 9, Y = 27.64 and Z = −11.206 with dimensions for SARS-CoV-2 M^pro^. Its size was set to 60 × 50 x 50 Angstroms to cover the active site. The structure of Apigenin and Apigenin analogues were downloaded from the PubChem search (https://pubchem.ncbi.nlm.nih.gov/). AutodockVina program was performed between Apigenin analogues and SARS-CoV-2 M^pro^ for molecular docking analysis such as binding types of interactions, binding energies, inhibition activities, ligand efficiency and distances. Molecular docking scores were set as AutoDock tools of the molecular graphics laboratory software package by keeping the analogue flexible [40]. Boceprevir was used as control to compare the molecular docking results with the Apigenin analogues.

### 2.3. LigPlot analysis

Academic licensed LigPlot software was obtained from https://www.ebi.ac.uk/. This program is used to provide 2-D representation of protein–ligand interactions, intermolecular interactions like hydrogen bonding, hydrophobic interactions and atom accessibilities of their strengths [41].

### 2.4. In-silico Osiris/Molinspiration and ADMET analysis

Osiris and Molinspiration analyses are performed to describe 2D models and to indicate the type of pharmacophore site [42,43]. These analyses are employed to predict pharmacophore site and biological activity of the apigenin analogues and to determine the drug-likeness score. The acute toxicity in rodent models and chemical classification of the test compounds were predicted by GUSAR [44]. It analyzes compounds based on the quantitative neighborhoods of atom descriptors and prediction of activity spectra for substance algorithm and correlates the obtained results with the SYMYX MDL toxicity database. Furthermore, it classifies them based on the Organization for economic co-operation and development (OECD) chemical classification manual. The pharmacokinetic properties of the apigenin analogues were achieved with using the SwissADME, which is an open online tool (http://www.swissadme.ch). The ADME properties define blood–brain barrier (BBB) permeability and passive human gastrointestinal absorption (HIA) as well as substrate or nonsubstrate permeability glycoprotein (P-gp) and cytochrome P450 (CYP) [42].

## 3. Results

‘This study aims to investigate the virus mutations and/or antigenicity changes and find the conserved targets. According to molecular and modeling studies, we confirm the genetic stability of SARS-CoV-2. Main proteases from different variant and that it constitutes a high-profile drug target. For faster and more cost-efficient drug discovery, we used the *in-silico* approach for prediction of the inhibitory activities of apigenin and its analogues against this target. The study confirms the potential of the apigenin 7-glucoside-4’-p-coumarate to inhibit M^pro^ SARS-CoV-2. It was observed that this analogue obtained good pharmacokinetic and toxicological characteristics. These finding suggest the ability to substitute boceprevir by this natural product present in several local plants for SARS-CoV-2 treatment.’

### 3.1. Conserved sequence among M^pro^ SARS-CoV-2

**Figure 1.**
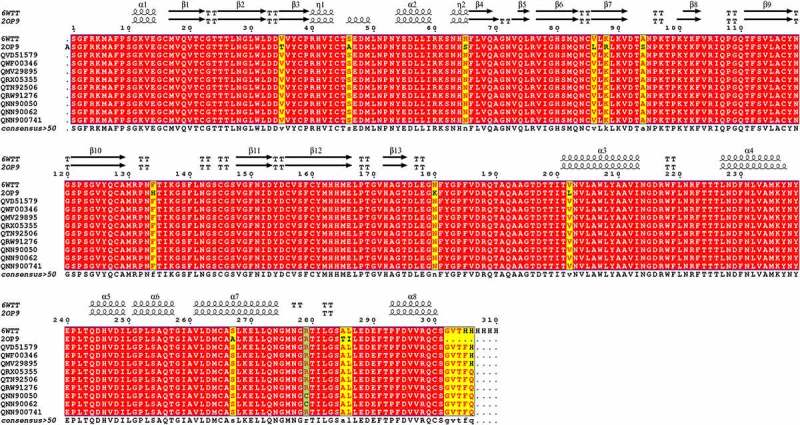
Structure-based multiple sequence alignment of SARS-CoV-2 M^pro^ from different variant (6WTT, 2OP9, QVD51579.1, QWF00346.1, QMV29895.1, QRX05355.1, QTN92506.1, QRW91276.1, QNN90050.1, QNN90062.1 and QNN90074.1). Residues invariable among sequences are typed in white on a red background; residues conserved within each group are typed in red on a yellow background. The residue mutated in this study (R279) is indicated in light blue. Secondary structure elements from of M^pro^ structure are indicated at the top of the alignment with SARS-CoV-2 main proteases (PDB code: 6WTT) .

A thorough comparison of the primary and secondary structures of the 3 CL protease sequence (M^pro^) was carried out (Figure 1). The alignment of 10 sequences of M^pro^ from different variant of SRARS-Cov-2 and the sequence of M^pro^ from SARS-CoV (2OP9 [45]) showed the presence of 12 mutations (Identity = 93.23%). However, all SARS-CoV-2 M^pro^ sequences are 100% conserved except for two Tunisian variants showing only 1 residue of 306 different from that of SARS-CoV-2 (identity = 97.74%) (Figure 1).

### 3.2. Structural aspects

**Figure 2.**
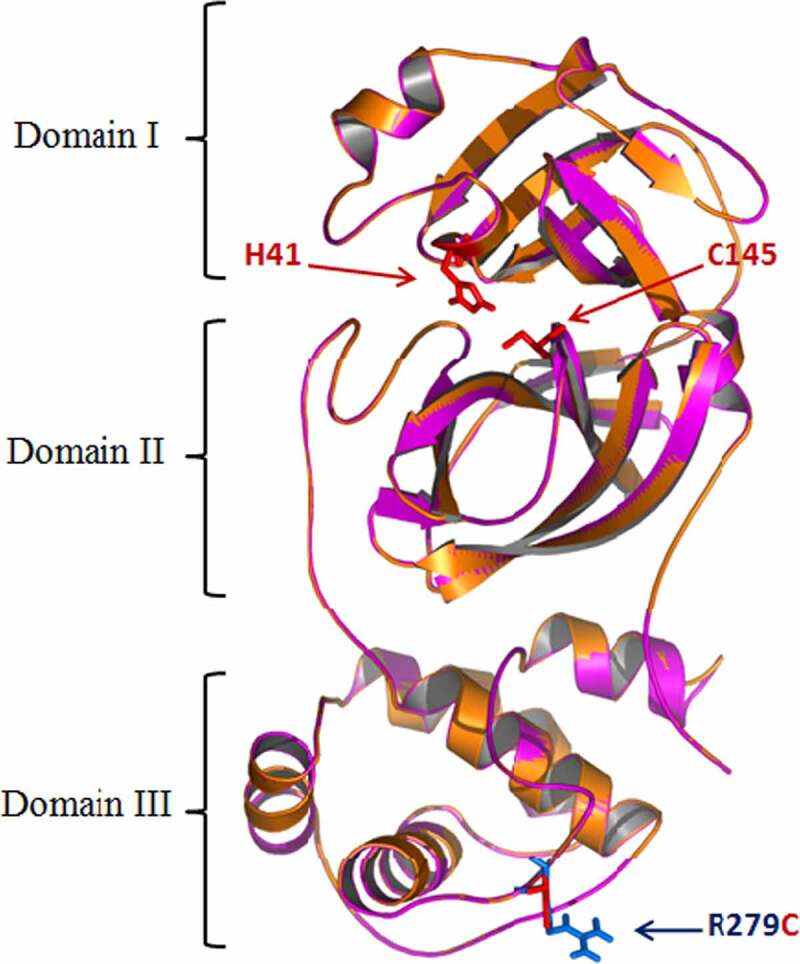
Superimposition of structure of the SARS-CoV-2 M^pro^ (pdb: 6WTT) (Orange) and model of the SARS-CoV-2 3 M^pro^ fromTunisian variant QNN90062.1 (pink). The catalytic dyad (Cys-145 and His-41) is colored in red.

The modeling of SARS-CoV-2 Main proteases from different variants and the characterization of the mutations structural impacts was investigated. M^pro^ model (306 residues) was built by the aid of the automated homology modeling, Geno3D, web server using SARS-CoV-2 Main protease (PDB ID: 6WTT, chain A) as homolog. The M^pro^ sequence exhibited high identity with that of the template (99.76%) suggesting the high-quality models that could be obtained. The obtained model showed a perfect superimposition of the Cα with 6WTT regarding to the very low RMSD (root mean square deviation) value estimated at 0.914 Å. The Main protease of SARS-CoV-2 was showed as composed of three domains (Figure 2). The domains I and II have an antiparallel β-barrel fold. The cleft between these domains generates the substrate-binding site. Domain III has a globular structure formed by five α-helices. A loop (residues 183–198) connected this domain to domain II. M^pro^ SARS-CoV-2 has a C145-H41 catalytic dyad. Modeling results showed that the mutation (R279C) is situated in domain III, far of the active site (Figure 2). These data confirmed that the structure is similar to main protease of SARS-CoV-2 [46] and the genetic stability of main proteases of SARS-CoV-2 from different variants.

### 3.3. Molecular docking studies

**Figure 3.**
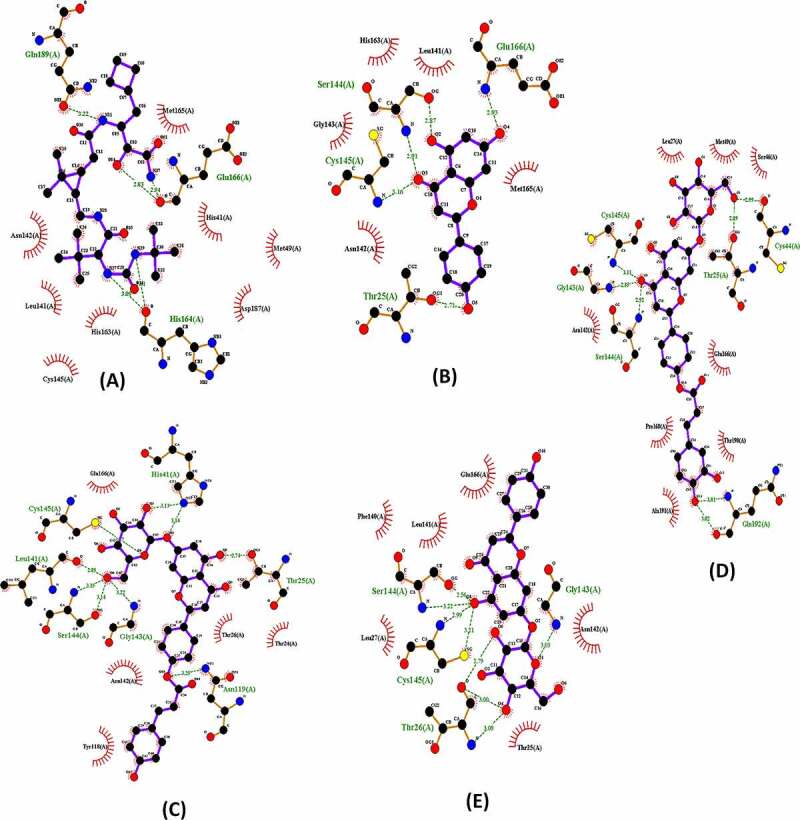
Interactions of the SARS-CoV-2 M^pro^ (pdb: 6WTT) with Boceprevir (a), Apigenin (b), Apigenin 7-glucoside-4’-p-coumarate (c), Apigenin 7-glucoside-4’-trans-caffeate (d) and Apigenin 7-O-beta-D-glucoside (Cosmosiin) (e) .

In this study, the four Apigenin analogues along with the Boceprevir were investigated as potential inhibitors of the M^pro^ SARS-CoV-2 using Autodock Vina tools. Boceprevir was showed, using enzyme inhibition and co-crystal structure analyses, to inhibit replication of SARS-CoV-2 in cell culture [11,47]. In this study, all Apigenin analogues as well as Boceprevir were docked using similar optimized docking conditions. All the docked poses into the binding site of M^pro^ SARS-CoV-2 were analyzed with identified docking search algorithms and scoring functions (Table 1 and Figure 3).

**Table 1.** Details of the Apigenin analogues compounds and Boceprevir from the docking analysis with Mpro substrate binding site

Compounds	Binding energies (Kcal/mol)	Interactions of the docked compounds to M^pro^	H-bonds interaction	Vander Waals interaction
Boceprevir	−6.6	Figure 3a	Gln189 Glu166(2) His164(2)	Met165 His41 Met49 Asp187 His163 Cys145 Leu141 Asn142
Apigenin	−7.2	Figure 3b	Glu166 Thr25 Cys145 Ser144(2)	Met165 Leu141 His163 Gly143 Asn142
Apigenin 7- glucoside- 4’- p-coumarate	−8.8	Figure 3c	His41(2) Cys145 Leu141 Ser144(2) Gly143 Asn119	Glu166 Asn142 Tyr118 Thr26 Thr25
Apigenin 7-glucoside- 4’- trans-caffeate	−8.7	Figure 3d	Cys44 Cys145 Gly143 Ser144 Gln192(2) Thr25	Leu27 Met59 Ser46 Glu166 Thr190 Ala19 Pro168 Asn142
Apigenin 7- O- beta-D-glucoside (Cosmosiin)	−8.0	Figure 3e	Gly143 Ser144(2) Cys145(2) Thr26(3)	Glu166 Leu141 Phe140 Asn142 Leu27 Thr25

**Figure 4.**
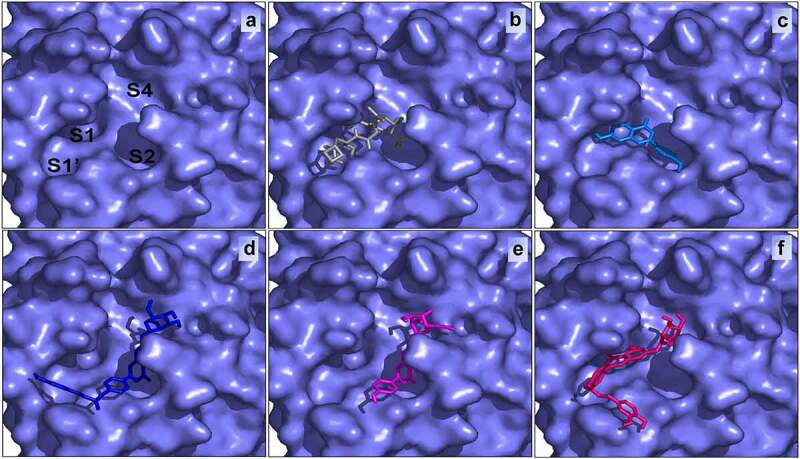
The active site of SARS-CoV-2 (a) bound with Boceprevir (b), Apigenin (c), Apigenin 7-glucoside-4’-p-coumarate (d), Apigenin 7-O-beta-D-glucoside (Cosmosiin) (e) and Apigenin 7-glucoside-4’-trans-caffeate (f) .

The poses made using Ligplot are shown in Table 1 for Boceprevir and the four ligands. Concerning Boceprevir, the best pose with the lowest binding energy (−6.6 Kcal/mol) showed that the hydroxyl and amide groups, resulting from the covalent addition to the α-ketoamide, form two hydrogen bonds with the main chain of Glu166. The tert-butyl group is relatively solvent exposed and forms two hydrogen bonds with His164. The amide bond on the main chain of Boceprevir forms hydrogen bond with the side chain of Gln189. Many other residues forming hydrophobic interactions (like His41, Met49, Met165, Asp163) stabilize the conformation of the ligand. Apart from our docked models, other published M^pro^ Docked results with boceprevir could be found [11,39]. These complex structures show highly similar binding poses to ours. However, structures containing Boceprevir are now available [47,48]. These complex structures show that the carbonyl of the electrophilic α-ketoamide could form a covalent bond with the sulfur of the catalytic residue Cys145 stabilizing the structure. The oxygen of the same group forms two hydrogen bonds with the main chain amides of Cys145 and Gly143. Hence, the oxyanion hole with its S1, S1’ and S2 pockets is occupied. Nevertheless, and as found by previous studies, the cyclobutylmethyl group of boceprevir is inserted superficially into the S1 pocket and is relatively solvent exposed (Figure 4).

According to Table 1, the best binding energy analogue was the Apigenin 7-glucoside-4’-p-coumarate (−8.8 kcal/mol) followed by the Apigenin 7-glucoside-4’-trans-caffeate (−8.7 kcal/mol). These two Apigenin analogues displayed with residues in active site of M^pro^ SARS-CoV-2, respectively, 9 and 7 hydrogen bonds.

For the Apigenin 7-glucoside-4’-trans-caffeate, among eight hydrophobic interactions, an amide π-stacked interaction (with Leu167) and a π-alkyl interaction (with Pro168) were established with the dihydroxyphenyl moiety of the caffeate. In addition, a π–π Tshaped was detected with His 41 established with the benzopyran ring of the Apigenin moiety. For the Apigenin 7-glucoside-4’-p-coumarate, five residues were found to be involved in hydrophobic interactions, and a π–π stacked interaction was detected between Tyr118 and the hydroxyphenyl ring of the coumarate moiety. Finally, concerning Cosmosiin (Apigenin 7-O-beta-d-glucoside), among other hydrophobic contacts, a π-sigma bond was established between Asn142 and benzopyran ring of the Apigenin. In all cases, the sugar moieties were involved essentially in hydrogen bonds.

### 3.4. Pharmacokinetic studies

The pharmacokinetic and toxicity properties of the Apigenin analogues with best binding energies were evaluated as potential drug candidates. General Unrestricted Structure–Activity Relationships (GUSAR) software [44] was used for quantitative *in-silico* toxicity prediction for Boceprevir, Apigenin and Apigenin analogues in rats with four types of administration (intraperitoneal, intravenous, oral and subcutaneous). As displayed in Table 2, the different LD50 values suggests that availability of the tested inhibitors for metabolism by the liver is a major factor for its toxicity. The LD50 value of Apigenin 7-glucoside-4’-p-coumarate and Cosmosiin was higher than Boceprevir for intravenous (IV), oral, and subcutaneous (SC) routes of administration. Only the LD50 value of Cosmosiin was higher through intraperitoneal (IP) route. By the OECD chemical classification system, only the Apigenin 7-glucoside-4’-trans- are class 4 when administered through Intraperitoneal compared to class 5 for the other compounds. The toxicity profile of these compounds is relatively low, and they require high doses to elicit toxic responses.

**Table 2.** Prediction of acute toxicity *in-silico* by GUSAR in rodent models and chemical classification of compounds

Ligands	Rat IP LD50 (mg/kg)	Rat IV LD50 (mg/kg)	Rat Oral LD50 (mg/kg)	Rat SC LD50 (mg/kg)	OECD Chemical Classification
Boceprevir	898.2	60.38	226.9	166.4	Class 5 in AD Class 4 in AD Class 3 in AD Class 4 in AD
Apigenin	689.1	22.7	896.6	5118.0	Class 5 in AD Class 4 in AD Class 4 in AD Non toxic in AD
Apigenin 7-glucoside-4’-p-coumarate	745.3	1108.0	3491.0	5174.0	Class 5 in AD Non toxic in AD Class 5 in AD Non toxic in AD
Apigenin 7-glucoside-4’-trans-caffeate	157.7	38.350	550.4	109.1	Class 4 in AD Class 3 in AD Class 4in AD Class 3 in AD
Apigenin 7-O-beta-D-glucoside (Cosmosiin)	1112.0	3790.0	2074.0	7941.0	Class 5 in AD Non toxic in AD Class 5 in AD Non toxic in AD

IP: Intraperitoneal; IV: Intravenous; LD50: Lethal dosage-50; OECD: Organization for economic co-operation and development; SC: Subcutaneous, in AD – compound falls in applicability domain of models.

The pharmacophore features and drug-like properties of the Apigenin and its analogues were performed with Molinspiration and Osiris Property Explorer [49]. The cLogP values (which is octanol/water partition coefficient) of the Apigenin analogues were found lower than 5.0 (Table 3). This finding suggests that these analogues have rational high absorption and permeability [50,51].

**Table 3.** Drug likeliness properties of the Apigenin analogues by swissAdme, osiris and Molinspiration

Drug likeness properties	Boceprevir	Apigenin	Apigenin 7-glucoside-4’-p-coumarate	Apigenin 7-glucoside-4’-trans-caffeate	Apigenin 7-O-beta-D-glucoside (Cosmosiin)
**Bioavaibility and** **drug-score^a^**					
Molecular weight g/mol	519.68	270.24	578.52	594.52	432.38
cLogP	1.38	2.34	2.11	1.76	0.35
Solubility	−4.02	−2.86	−4.58	−4.29	−2.74
TPSA	150.7	86.99	192.4	212.6	166.1
Druglikness	−8.76	1.21	−3.55	−2.7	−2.29
Drug-score	0.11	0.47	0.12	0.13	0.44
**Druglikness ^b^**					
GPCR ligand	0.50	−0.07	−0.04	−0.09	0.1
Ion channel modulator	0.04	−0.09	−0.49	−0.55	−0.01
Kinase inhibitor	−0.03	0.18	−0.12	−0.21	0.14
Nuclear receptor ligand	0.07	0.34	0.07	−0.02	0.31
Protease inhibitor	1.41	−0.25	−0.04	−0.06	0.02
Enzyme inhibitor	0.31	0.26	0.14	0.07	0.43
**Pharmacokinetics ^c^**					
GI absorption	Low	High	Low	Low	Low
BBB permeant	No	No	No	No	No
P-gp substrate	Yes	No	No	No	Yes
CYP1A2 inhibitor	No	Yes	No	No	No
CYP2C19 inhibitor	No	No	No	No	No
CYP2C9 inhibitor	No	No	Yes	Yes	No
CYP2D6 inhibitor	No	Yes	No	No	No
CYP3A4 inhibitor	Yes	Yes	No	No	No

GI: Gastrointestinal absorption; BBB: blood brain barrier; P-gp: Permeability Glycoprotein; CYP: Cytochrome P450; a = Osiris; b = Molinspiration; c = SwissAdme.

Solubility is known to be a significant parameter for drug design and pharmacology due to the potential absorption and distribution characteristics. Thus, soluble drugs are preferred in drug manufacturing [52]. The solubility values of most drugs sold in the market are greater than −4.0 and the solubility values of Apigenin and Cosmossin were −2.86 and −2.74, respectively. The solubility of Apigenin 7-glucoside-4’-p-coumarate and Apigenin 7-glucoside-4’-trans-caffeate values was also close to −4 (Table 3).

Furthermore, Ion Channel Modulator, Human G-protein coupled receptors (GPCRs) ligands, Nuclear Receptor Ligand, Kinase Inhibitor, Protease Inhibitor and Enzyme inhibitors of the Apigenin analogues were illustrated with the prediction bioactivity scores using online-site Molinspiration (Table 3). As presented in Table 3, metabolic enzymes such as Cytochrome P450 (CYP) and the transporter class P-glycoprotein (P-gp) were equally assessed in this study. Boceprevir, Apigenin 7-glucoside-4’-p-coumarate, Apigenin 7-glucoside-4’-trans-caffeate and Cosmossin were not found to be inhibitors of CYP except CYP2C9 inhibitor confirming the goodness of their transport in the intestine [53].

## 4. Discussion

The virus mutates and/or changes its antigenicity causing loss of the vaccination efficiency. Many experimental and computational efforts done to identify a genetic stable target for anti-SARS-CoV drug design were provided. In this work, the genetic stability of Main proteases of SARS-CoV-2 from different variants was confirmed. In addition, the superimposition of the crystal structures of M^pro^ with and without ligands has RMSD ranging from 0.26 to 0.38 Å, indicated that the binding pocket is pre-shaped [54]. Thus, M^pro^ is an attractive target for anti-SARS-CoV drug design. The inhibitors of this enzyme can be found by structure-based design [35], by enzymatic assay of existing inhibitors of other virus main protease such as HIV or HVC protease inhibitors [55] or by screening of chemical database using docking approaches. Boceprevir is generally used as reference molecule especially for its low renal and hepatic toxicity [56]. The recent reports suggest that boceprevir inhibits the enzymatic activity of M^pro^ with an IC50 value of 4.13 μM [55]. This result was confirmed by co-crystallization of M^pro^ and Boceprevir [57].

In order to find natural molecules as an alternative of Boceprevir, the Apigenin and Apiginin analogues were investigated as potential inhibitors of the M^pro^ SARS-CoV-2. The docking results showed that the binding energy values (−8.8 to −7.2 kcal/mol) are better than that of Boceprevir and two Boceprevir analogues (PubChem ID 57841991 and 58,606,278) with binding energies of −6.6, −7.2 and −7.5 kcal/mol, respectively [11].

Apigenin 7-glucoside-4’-p-coumarate occupies the S1′, S2, and S4 subsites while Boceprevir filled the S1, S1′, and S2 subsites of SARS-CoV-2 M^pro^ active site. Generally, small size inhibitors may bind only at S1 and S2 subsites [11,58]. Boceprevir with its medium size may bind to S1′, S1, and S2 subsites [59,60], while some large inhibitors may bind to S1’, S1, S2, and may extend through S3 subsites as for example alpha-ketoamide (PDB: 6Y2F) [61,62]. These compounds have dissimilar structures and sizes, yet they bind and inhibit the activity of SARS-CoV-2 M^pro^.

All hydrogen bonds and hydrophobic interactions are shown in Table 1. The common residues in active site of M^pro^ SARS-CoV-2 stabilizing all analogues best poses are His 41, Ser144, Gly143, Thr25, Thr26, Glu166, Asn142 and especially the catalytic Cys145. The latter residue, because of its proximity to the ligand, was also suspected to establish covalent bond with certain atoms thanks to its thiol group as it has been demonstrated in the crystallization experiments but not been detected by docking experiments [32]. All the aforementioned residues are identified as key interactions between SARS-CoV-2 main protease and inhibitor drug candidates [34].

We observed for all Apigenin analogues strong hydrogen bonding. In addition, although the thiol group of Cys145 which was found to interact via hydrogen bonds, it is suspected to be able for covalent bonds as confirmed by previous structural studies. Gly143, Ser144, Glu166, Asn142 and Cys145 also interact with each inhibitor and are probably implicated in its stabilization. The best binding energy molecule such as Apigenin 7-glucoside-4’-p-coumarate occupies the S1′, S2, and S4 subsites (Figure 3).

Apigenin 7-glucoside-4’-p-coumarate was found to have the best desirable pharmacokinetic properties such as low hepatotoxicity, good aqueous solubility, high intestinal absorption, non CYP2D6 binding and inability to cross the BBB besides good binding properties with SARS-CoV-2 M^pro^ active site. Therefore, we demonstrate that the Apigenin 7-glucoside-4’-p-coumarate identified by our *in-silico* study have potential against COVID-19 and may bind and inhibit the SARS-CoV-2 main protease.

Similar studies on structure–activity relationship analysis demonstrated that polyphenols and flavonoid (naringenin, rutin and tangeretin) from *Citrus* and *Curcuma* spp. have high affinity to the active site of the M^pro^ [63].

Natural plant medicines are shown to ameliorate the recovery of infected person and to prevent SARS-CoV-2 infection of healthy persons as well as to improve the health state of patients with mild or severe symptoms [64]. Among others, plant originated cinnamic amides, flavonoids, chalcones, tanshinones and diarylheptanoids are shown to inhibit the PLpro one of the nonstructural proteins encoded by the SARS-CoV-2 genome [65]. However, other natural products are demonstrated to inhibit the 3 CL(pro), another nonstructural protein of the SARS-CoV-2. As examples alkylated Chalcones, phlorotannins and bioflavonoids were described in the literature [66]. Most of the studies were performed using *in-silico* approaches and few of them combined in vitro and/or in vivo experiments. Most of the studies were conducted with isolated natural compounds and phenolic compounds were the most frequently reported. In this line, our study confirmed the potential of the Apigenin 7-glucoside-4’-p-coumarate to inhibit Mpro SARS-CoV-2. It was observed that this analogue obtained good pharmacokinetic and toxicological characteristics. These finding suggest the ability to substitute Boceprevir by this natural product present in several local plants for SARS-CoV-2 treatment. However, the *in-silico* study evaluating the anti-SARS-CoV-2 effect is still insufficient even if this natural products could be considered as promising anti-SARS-CoV-2 agents.

## 5. Conclusion

Our study confirmed the potential of the Apigenin 7-glucoside-4’-p-coumarate, naturally present in different Tunisian plants, to inhibit M^pro^ SARS-CoV-2 with the best binding energy. It was observed that this analogue obtained good results in terms of its toxicity properties. These finding suggest the ability to substitute Boceprevir by apigenin 7-glucoside-4’-p-coumarate for SARS-CoV-2 treatment.

## Highlights

- Main protease of SARS-CoV-2 (M^pro^) is a high-profile drug target.
- Good pharmacokinetic and toxicological characteristics of Apigenin analogues.
- Apigenin 7-glucoside-4’-p-coumarate may be a good candidate for M^pro^ inhibition.
